# miR-340 and ZEB1 negative feedback loop regulates TGF-β- mediated breast cancer progression

**DOI:** 10.18632/oncotarget.8421

**Published:** 2016-03-27

**Authors:** Li-Kun Hou, Yue Yu, Ye-Gong Xie, Jie Wang, Jie-Fei Mao, Bin Zhang, Xin Wang, Xu-Chen Cao

**Affiliations:** ^1^ The First Department of Breast Cancer, Tianjin Medical University Cancer Institute and Hospital, National Clinical Research Center for Cancer, Tianjin 300060, China; ^2^ Key Laboratory of Cancer Prevention and Therapy, Tianjin 300060, China; ^3^ Key Laboratory of Breast Cancer Prevention and Therapy, Tianjin Medical University, Ministry of Education, Tianjin 300060, China

**Keywords:** miR-340, ZEB1, TGF-β, EMT, breast cancer

## Abstract

MicroRNAs act as key regulators in carcinogenesis and progression in various cancers. In present study, we explored the role of miR-340 in the breast cancer progression. Our results showed that overexpression of miR-340 inhibits breast cancer cell proliferation and invasion, whereas depletion of miR-340 promotes breast cancer progression. Molecularly, ZEB1 was identified as a target gene of miR-340 and miR-340 suppressed the expression of ZEB1 by directly binding to the 3′-UTR of ZEB1. Furthermore, ZEB1 transcriptionally suppresses miR-340 expression. The negative feedback loop regulated TGF-β-mediated breast cancer progression. In conclusion, our data suggested that miR-340 acted as a tumor suppressor in breast cancer progression.

## INTRODUCTION

Breast cancer is one of the most dangerous diseases for women worldwide. In China women, it is the most common cancer and the third most common cause of cancer-related death [1]. In China, the number of women suffering from breast cancer shows a trend to increase while the number of death tends to decrease in future [2]. Although improvements in early detection and treatment have decreased the mortality rates of breast cancer in recent years, prevention and therapy of breast cancer remain a major public health concern [3]. Thus, identification and determination of new genes/pathways involved in breast cancer carcinogenesis will help to develop safer and faster diagnosis and better disease prognosis predication following treatment of this disease.

MicroRNAs (miRNAs) are a class of short (18~22 nucleotides), single-stranded, non-coding RNA sequences which could regulate gene expression at post-transcriptional level. They bind to the 3′ untranslated region (UTR) of their target mRNAs, modulating mRNA stability and/or translation [4–6]. Up to now, many miRNAs have been reported to be related to various cancer carcinogenesis and progression including breast cancer [7–11]. It has been confirmed that miR-340 are differentially expressed between BC patients with metastasis versus these without metastasis and miR-340 are implicated in the status of BRCA1/2 in BC patients [12–14].

Epithelial-mesenchymal transition (EMT) is defined as a process that specific cells change their phenotype from epithelial to mesenchymal, that is, polarized immotile epithelial cells to motile mesenchymal cells. It will lead to increased motility and invasion. So EMT is involved in not only embryonic development but also malignant progression [15–17]. Zinc finger E-box binding homeobox 1 (ZEB1) is an important EMT transcription factor conducting tumor metastasis. ZEB1 is a driver of the EMT branch of epithelial plasticity, and it is a potential prognostic marker in a lot of cancers. ZEB1 was proved to be related to tumor genesis and metastasis [18–21]. It's well known that ZEB1 and miR-200 family have a double-negative feedback loop. ZEB1 is also a target for some other miRNAs [22, 23]. The role of miR340 in regulating cell invasion and the interaction between miR-340 and ZEB1 are still to be explored.

In the present study, we investigated the effect of miR-340 on the breast cancer progression, and the relationship between miR-340 and ZEB1. What we found may provide a new prognostic marker or therapeutic target for breast cancer patients.

## RESULTS

### miR-340 suppresses breast cancer cell proliferation and invasion *in vitro*

**Figure 1:**
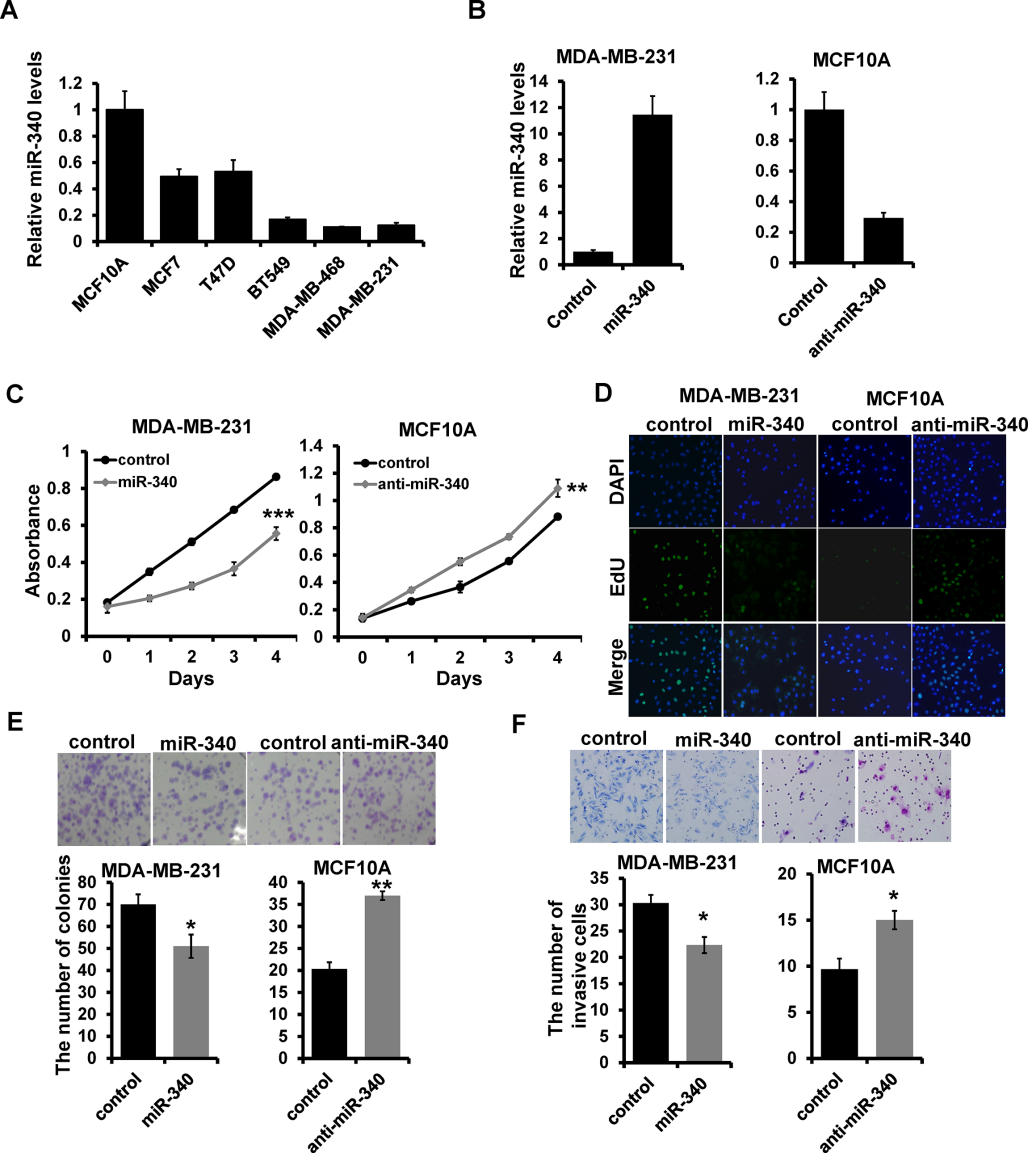
The effect of miR-340 on proliferation and invasion in breast cancer cell (A) miR-340 expression in the indicated breast cancer cell lines with respect to the expression in breast epithelial cell line MCF10 (**A** and **B**) The miR-340 expression in miR-340 mimic-transfected MDA-MB-231 and miR-340 inhibitor-transfected MCF10A cells by qRT-PCR. (**C, D, E**) The ability of cell proliferation measured by MTT (C), EdU (D) and colony formation (E) assays. (**F**) Transwell analysis of cell invasion. *P < 0.05, ***P < 0.001.

To investigate the role of miR-340 in breast cancer development and progression, we examined its expression in five different breast cancer cell lines (MCF7, T47D, BT549, MDA-MB-468, and MDA-MB-231) and breast epithelial cell line (MCF10A) by RT-qPCR. We observed that miR-340 is down-regulated in all breast cancer cell lines compared with that of MCF10A (Figure 1A). To determine the influence of miR-340 in breast cancer progression, we transfected the corresponding miR-340 mimic in MDA-MB-231 cells while inhibitor in MCF-10A cells which confirmed by RT-qPCR (Figure 1B). Overexpression of miR-340 decreased the ability of cell proliferation compared with the mimic control cells by MTT (Figure 1C; left), Edu (Figure 1D; left) and colony formation (Figure 1E; left) assays. While depletion of miR-340 increased the ability of cell proliferation (Figure 1C, 1D and 1E; right). Transwell assays showed that miR-340 overexpression inhibited the invasive ability of MDA-MB-231 cells, whereas depletion of miR-340 promoted the cell invasion (Figure 1F). Together, these results indicated that miR-340 suppresses breast cancer progression *in vitro*.

### miR-340 inhibits EMT in human breast cancer cells

**Figure 2:**
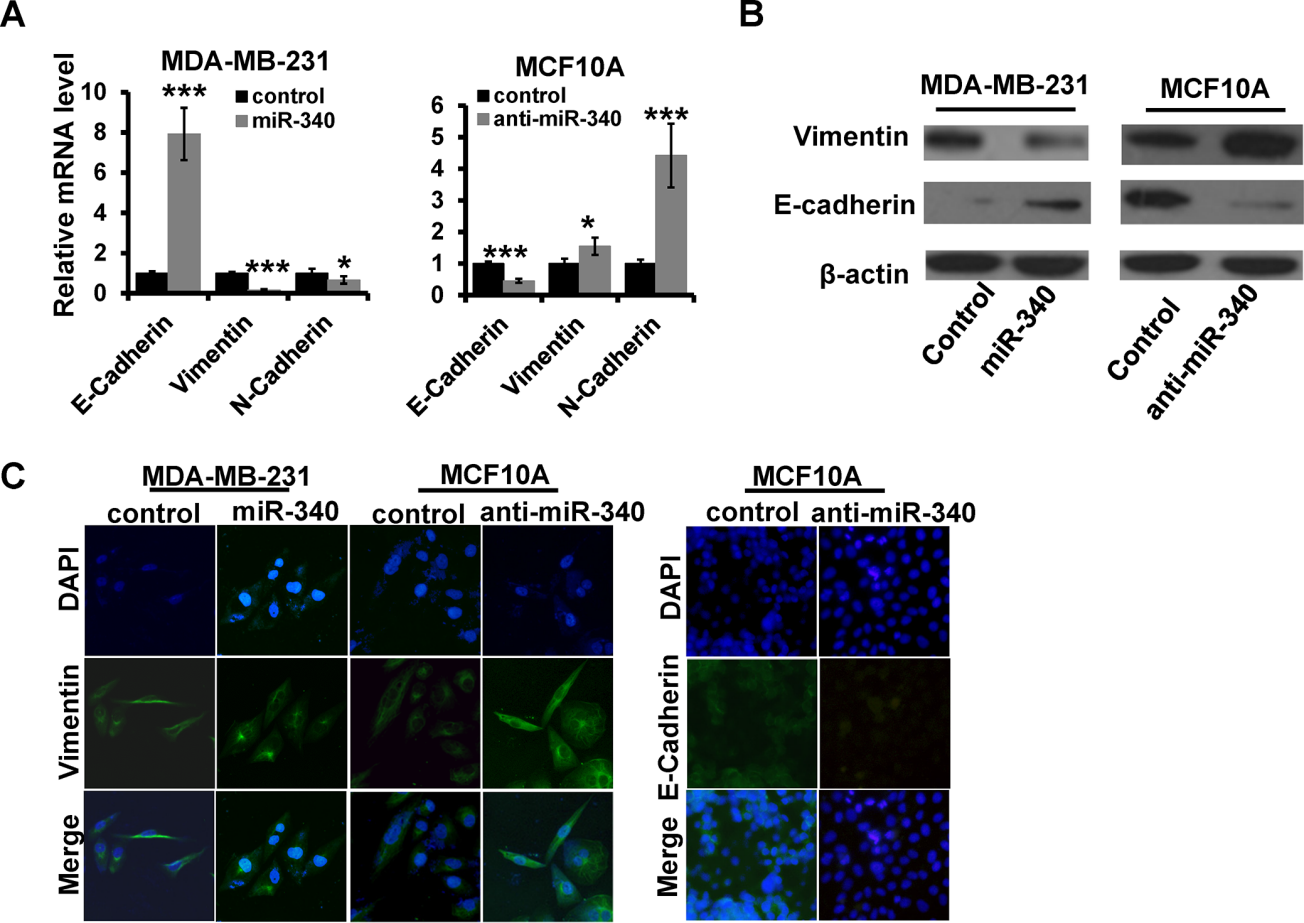
The effect of miR-340 on the expression of EMT markers (**A** and **B**) The mRNA and the protein expression levels of EMT markers detected in indicated cells by RT-qPCR (A) and western blot (B). (**C**) The expression of E-cadherin and Vimentin detected by immunofluorescence. *P < 0.05, ***P < 0.001.

Next, we explored the role of miR-340 in breast cancer EMT. We found that overexpression of miR-340 could reduce the expression of mesenchymal phenotypic markers, including Vimentin, N-cadherin, while increase the expression of epithelial phenotypic markers, including E-cadherin in MDA-MB-231by RT-qPCR (Figure 2A; left) and western blot (Figure 2B; left) assays. Meanwhile, depletion of miR-340 resulted in up-regulated expression of Vimentin and N-cadherin, and down-regulation of E-cadherin (Figure 2A and 2B; right). Immunofluorescence staining analysis further identified that the expression of vimentin was decreased in miR-340-overexpressed MDA-MB-231, whereas was increased in miR-340-depleted MCF10A compared with control cells. Moreover, the expression of E-cadherin was increased in miR-340-depleted-MCF10A cells (Figure 2C). Thus, these results showed that miR-340 inhibits EMT in breast cancer cells.

### ZEB1 is a direct target of miR-340

**Figure 3:**
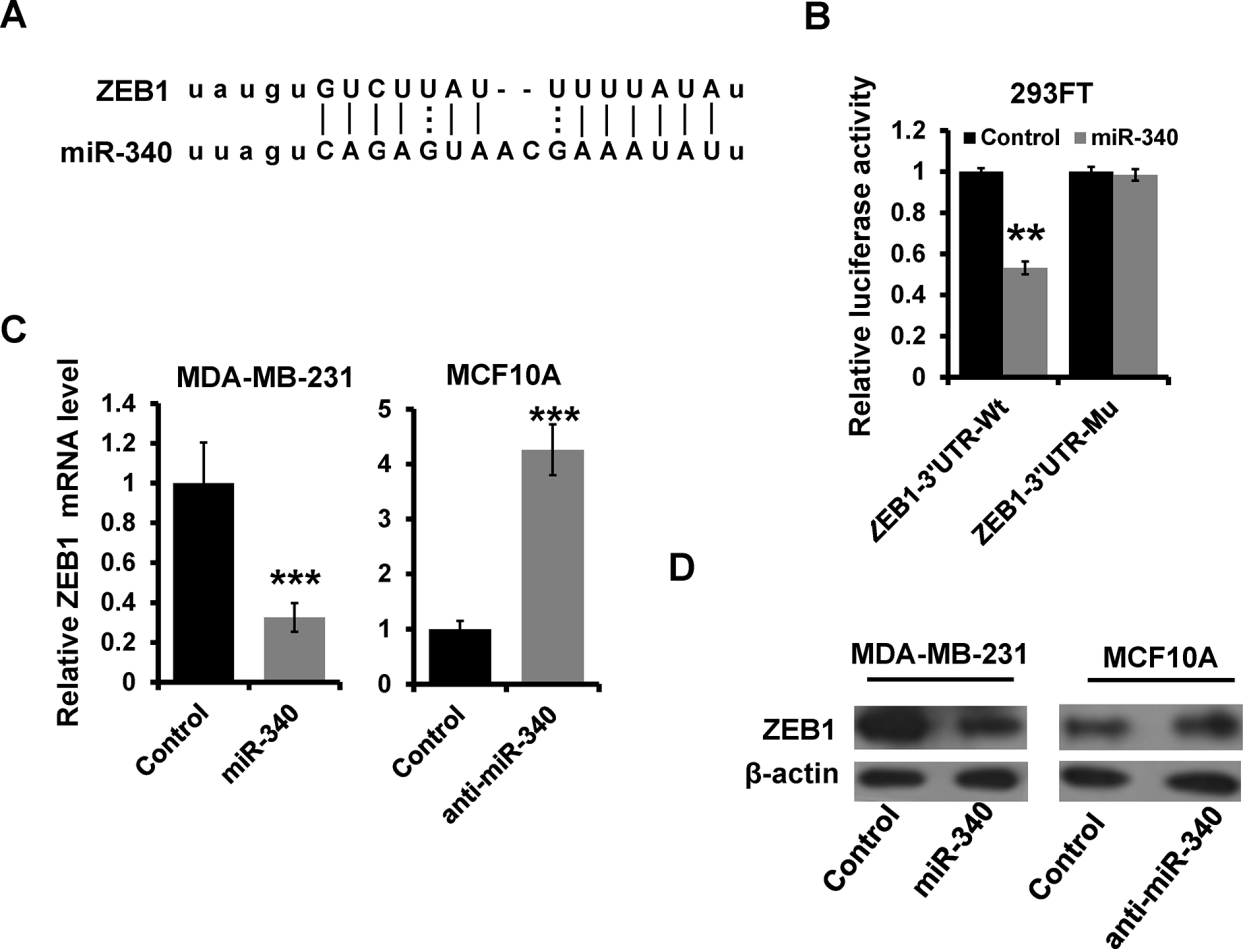
ZEB1 is a direct target of miR-340 (**A**) Schematic illustration of the predicted miR-340-binding site in ZEB1 3′-UTR. (**B**) Luciferase reporter analysis was performed to validate miR-340 target ZEB1. A 3′UTR fragment containing the predicted miR-340 targeting site of ZEB1 was fused downstream of the Luc gene in pGL3-control plasmid (ZEB1–3′UTR-wt). A miR-340 mutated binding site was also constructed (ZEB1–3′UTR-mu). (**C** and **D**) The mRNA and the protein expression levels of ZEB1 in indicated cells by RT-qPCR (C) and western blot (D). ***P < 0.001.

Based on the miR-target analysis using (http://www.microrna.org) [24], we thought that ZEB1 was a potential target gene of miR-340. The predicted binding sites on miR-340 with ZEB1 3′UTR was showed in Figure 3A. We constructed a luciferase reporter plasmid containing the 3′ UTR of ZEB1 and examined the luciferase activities. As shown in Figure 3B, overexpression of miR-340 decreased the luciferase activity of the ZEB1 3′-UTR in HEK 293 cells (Figure 3B). In addition, site-directed mutagenesis of the seed region abolished the inhibitory effect of miR-340 on luciferase activity (Figure 3B). We further observed that the expression of ZEB1 mRNA and protein was decreased in miR-340-overexpressed MDA-MB-231 cells, meanwhile the expression was increased in miR-340-depleted MCF10A cells by RT-qPCR (Figure 3C) and western blot (Figure 3D) assays. Thus, these results indicated that ZEB1 is a target gene of miR-340 in breast cancer cells.

### ZEB1 directly suppresses miR-340

**Figure 4:**
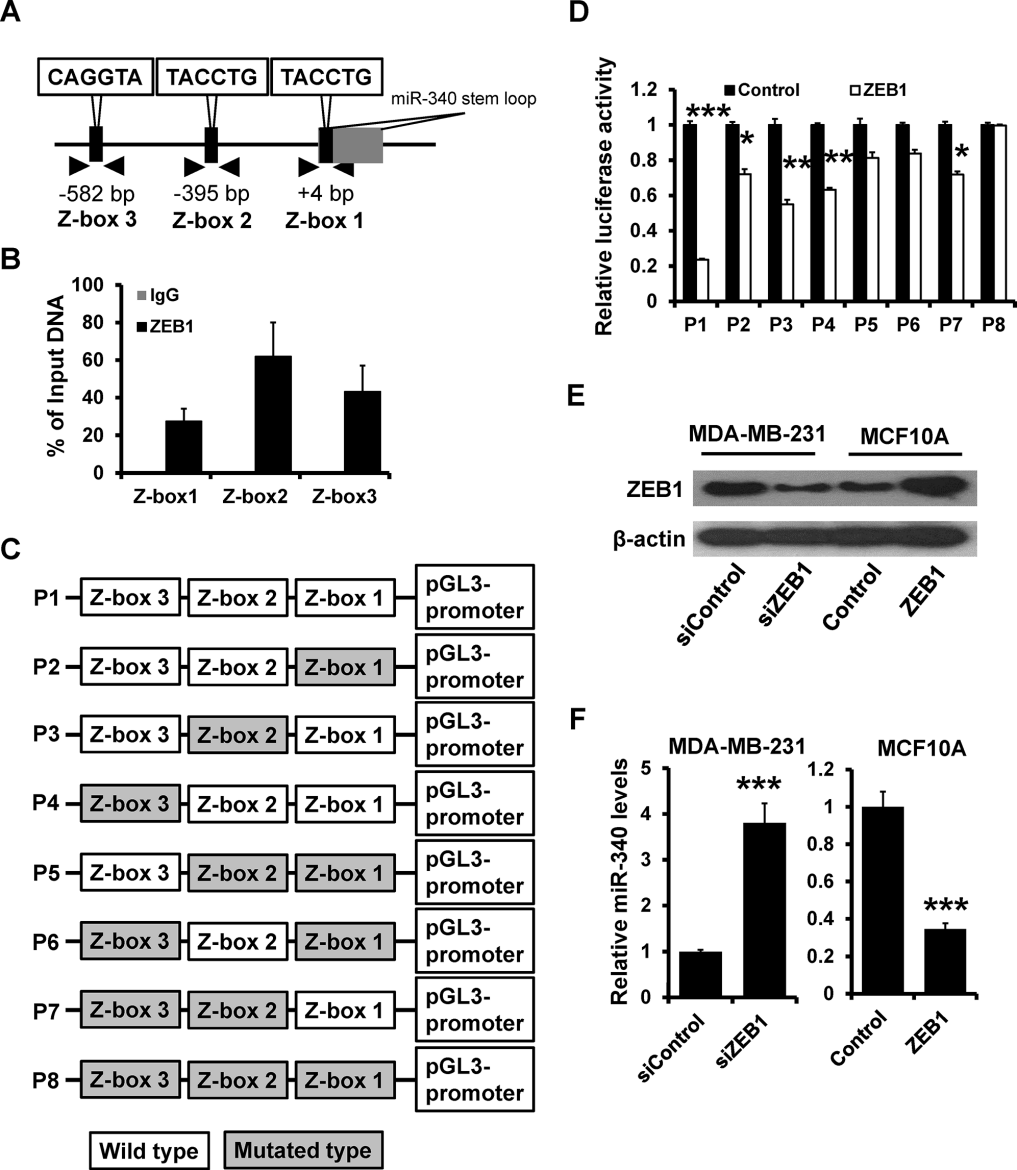
ZEB1 directly suppresses miR-340 expression (**A**) Schematic illustration of the Z-boxes in upstream of miR-340 promoter. (**B**) ChIP assay was performed to confirm the combination between ZEB1 and miR-340 promoter. (**C**) Schematic illustration of the pGL3-promoter plasmid which contain the wild type and the mutated type of Z-box sites. (**D**) The luciferase activity were detected after transfected with ZEB1. (**E**) The level of ZEB1 was confirmed by western blot after transfected with ZEB1 siRNA and ZEB1. (**F**) The level of miR-340 detected by RT-qPCR after transfected with ZEB1 siRNA and ZEB1. *P < 0.05, **P < 0.01, ***P < 0.001.

We next analyzed the putative promoter of miR-340 and found three putative binding sites which were restricted to ZEB factors (Z-box 1 to 3, CAGGTA; Figure 4A). ChIP assays showed that ZEB1 could bind to all the three Z-boxs (Figure 4B). After cloning of the wild (P1) or Z-box-mutated (P2 to P8) putative promoter into a luciferase reporter vector (Figure 4C), we found that the wild type promoter activity was repressed by overexpression of ZEB1. However, when all of Z-boxes were mutated, the activity was not affected by ZEB1 overexpression in HEK 293 cells (Figure 4D). After transfected with ZEB1 siRNA and pcDNA3.1-ZEB1 in MDA-MB-231 and MCF-10A (Figure 4E), the level of miR-340 has a markedly change. The down-expression of ZEB1 led to the increase of miR-340, while the overexpression of ZEB1 reduced the level of miR-340 expression (Figure 4F). Together, these results showed that ZEB1 could directly suppresses miR-340 expression.

### miR-340 inhibits breast cancer progression by regulating ZEB1

**Figure 5:**
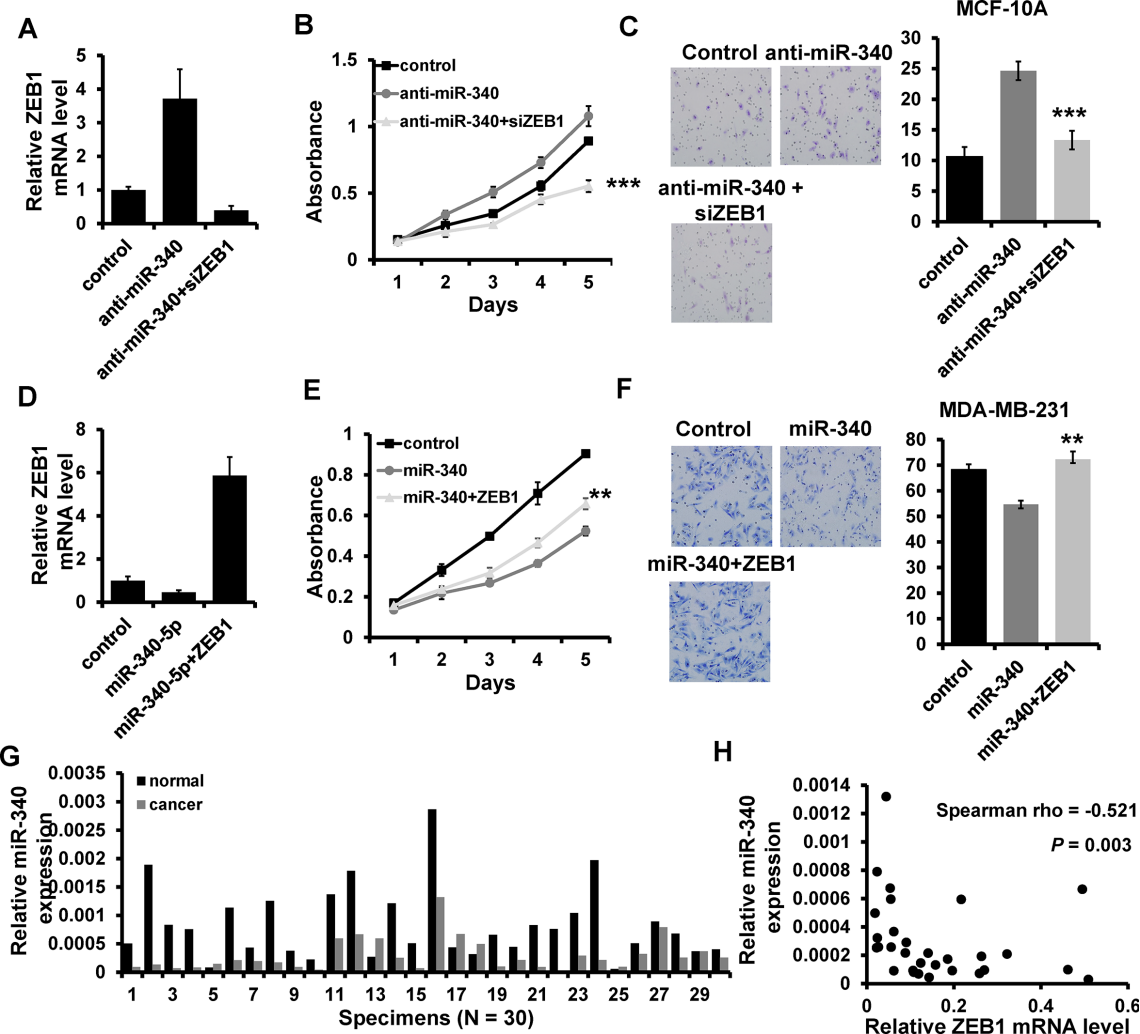
miR-340 regulates breast cancer progression through regulating ZEB1 (**A** and **D**) The mRNA level of ZEB1 detected by RT-qPCR in indicated cells. (B), (**E**) The ability of cell proliferation in indicated cells measured by MTT. (C), (**F**) Transwell analysis of cell invasion in indicated cells. (**G**) The expression level of miR-340 in breast cancer tissues and the paired normal breast tissues. (**H**) miR-340 is associated with ZEB1 expression in breast cancer tissues. **P < 0.01, ***P < 0.001.

To further confirm the regulation of ZEB1 by miR-340, we performed a series of rescue experiments. pcDNA3.1-ZEB1 was co-transfected with miR-340 mimic into MDA-MB-231, whereas ZEB1 siRNA was co-transfected with miR-340 inhibitor into MCF10A. The expression of miR-340 and ZEB1 was confirmed by RT-qPCR (Figure 5A and 5D). The results showed that overexpression of ZEB1 almost entirely reverses the inhibition of miR-340 on cell proliferation and invasion (Figure 5E and 5F). Moreover, the ability of proliferation and invasion was significantly abolished by ZEB1 siRNA in MCF10A (Figure 5B and 5C). We next examined the miR-340 expression in 30 cases of primary breast cancer tissues and the paired normal breast tissues by RT-qPCR. The results showed that miR-340 expression is down-regulated in breast cancer tissues compared the paired normal breast (Figure 5G). Moreover, the miR-340 expression level was negatively related to the ZEB1 mRNA level (Figure 5H). Together, these results showed that miR-340 inhibits breast cancer progression by regulating ZEB1.

### TGF-β signaling was involved in the feedback loop between miR-340 and ZEB1

**Figure 6:**
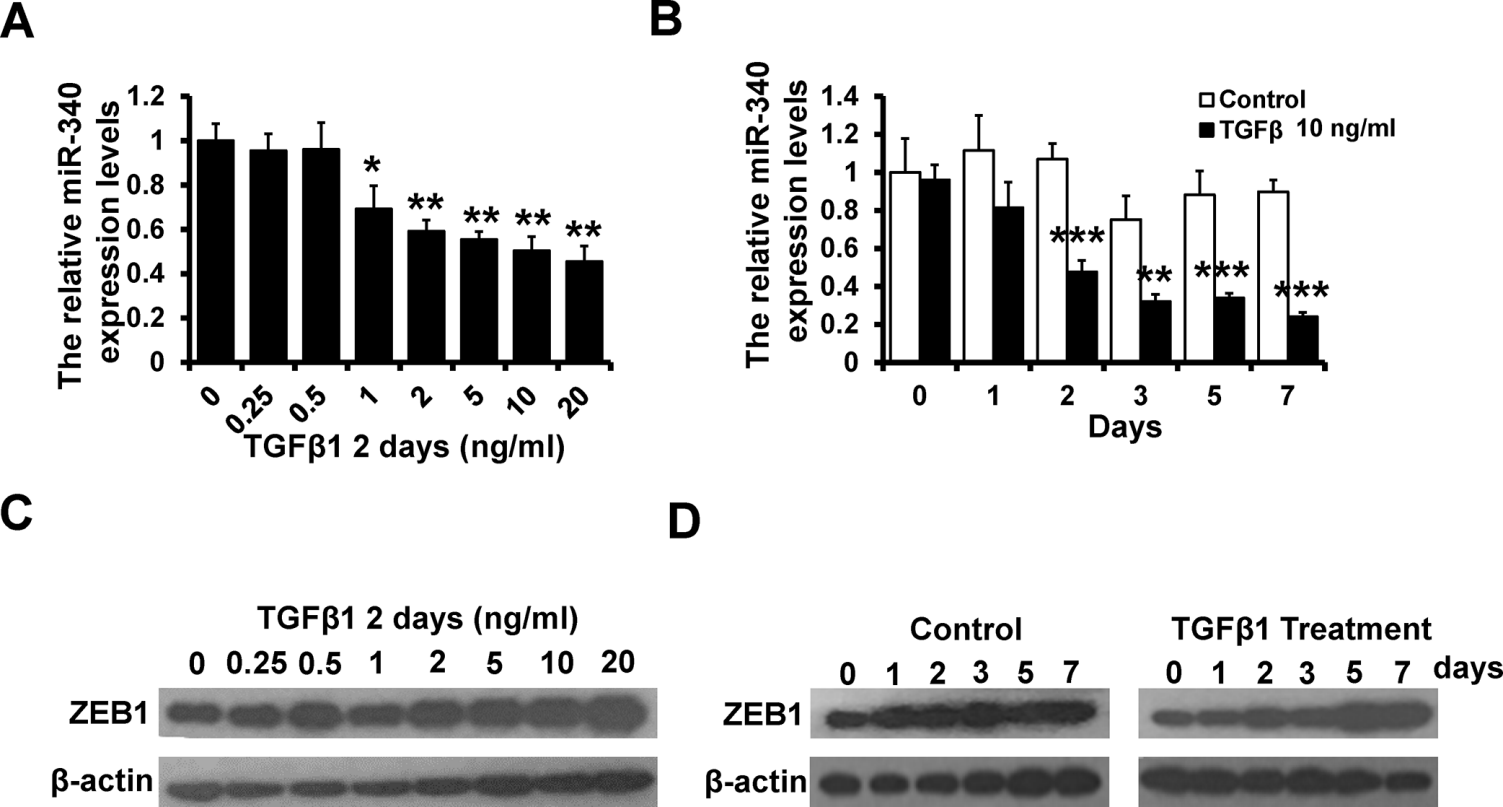
TGF-β signaling was involved in the feedback loop between miR-340 and ZEB1 (**A**) The level of miR-340 detected by RT-qPCR after stimulated with TGF-β1 in different concentrations for 2 days in MCF10A cells. (**B**) The level of miR-340 detected by RT-qPCR after stimulated with TGF-β1 (10 ng/ml) for 7 days in MCF10A cells. (**C**) The protein level of ZEB1 detected by western blot after stimulated with TGF-β1 in different concentrations for 2 days in MCF10A cells. (**D**) The protein level of ZEB1 detected by western blot after treatment with TGF-β1 for 7 days in MCF10A cells. *P < 0.05, **P < 0.01, ***P < 0.001.

Transforming growth factor (TGF)-β signaling is important for EMT and the expression of ZEB1 [25]. We stimulate MCF-10A cells with TGF-β1 in different concentrate for 2 days and 10 ng/ml TGF-β1 for 1~7 days. We observed that the level of miR-340 decreased along with the increase in the concentration and the time extension (Figure 6A and 6B). While the expression of ZEB1 increased with the TGF-β1 treatment in dose and time dependent manner (Figure 6C and 6D). Thus, these data indicated that the double-negative feedback loop between miR-340 and ZEB1 might achieve through the TGF-β signaling pathway.

## DISCUSSION

Sorts of miRNAs were known to be essential in tumor initiation and development, such as: miR-14, miR-181a, miR-361 [26–28]. Overexpression of miR-340 inhibits cancer cell proliferation and invasion [12, 14, 29–33]. Consistent with the study in breast cancer, we observed that miR-340 inhibit migration and invasion [12, 34]. However, they found that miR-340 did not affect the cell proliferation in MDA-MB-231. miR-340 inhibits tumor cell proliferation and induces apoptosis by inducing the stabilization of p27 in non-small cell lung cancer [29]. Through suppression of several oncogenes including p-AKT, EZH2, XIAP, CDK6, cyclin-D1 and cyclin-D2, overexpression of miR-340 inhibits cancer cell proliferation, migration and invasion, induces apoptosis and autophagy in glioblastoma and miR-340 is a marker for glioblastoma diagnosis and prognosis [30, 32, 33]. Moreover, evidences showed that miR-340 played an important role in regulating the liver metastasis by down-regulating c-met in colorectal cancer [31]. Consistent with their studies, we confirm that miR-340 act as a tumor suppressor in breast cancer progression.

Additionally, we found that miR-340 had an inverse effect on the EMT in the breast cancer cells. Cells which undergo a prototypical EMT often loss the delocalization of tight and adherens junction proteins, such as E-cadherin and claudins. Meanwhile, they assume a mesenchymal-like morphology with up-regulated expression of mesenchymal markers, such as N-cadherin and vimentin, and increased migratory and invasive properties [35, 36]. While the relationship between EMT and breast cancer metastasis is very close. Reports confirmed that cells tended to EMT might be the first step in the stromal invasion and metastasis of breast cancer, and occurrence of EMT in the breast tumor associated with high prevalence of CSCs, promoting tumor invasiveness and metastasis [37]. Therefore, we thought that miR-340 could suppress tumor progressive by suppression of EMT in breast cancer cells.

Furthermore, we determined that miR-340 directly targets the ZEB1 gene and showed that overexpression of miR-340 down-regulated the expression of ZEB1. Thinking about the relationship between ZEB1 and EMT, the results that miR-340 suppressed the markers of EMT were extremely reasonable. Because of the negative effect on E-cadherin, the overexpression of ZEB1 results in tumor metastasis and predicts an unpleasant prognosis in a lot of cancers, especially breast cancer [37, 38]. In bladder cancer, miR-23b inhibited cell proliferation and impaired colony formation by targeting ZEB1 directly [23]. These studies are consistent with our findings that miR-340 may have an effect on tumor growth and metastasis through targeting ZEB1.

Although the accurate mechanism how miRNAs regulated ZEB1 is not clear, ZEB1 has been identified as a target of several miRNAs. The most well-known miRNA regulating ZEB1 is miR-200 family. Studies showed that there was a double-negative feedback loop between ZEB1 and the miR-200 family [22]. What's more, miR-200c restored trastuzumab sensitivity in trastuzumab-resistant cells and suppressed invasion of breast cancer cells by silencing of ZEB1, ZNF217 or blockade of TGF-β signaling [39]. We found that miR-340 could regulate ZEB1 and be regulated by ZEB1. Moreover, TGF-beta signaling was involved in the relationship between miR-340 and ZEB1. What we found is highly consistent with the previous research indicating the loop between miR-340 and ZEB1.

## CONCLUSIONS

In conclusion, we demonstrated that the negative feedback loop of miR-340 and ZEB1 participates the breast cancer progression. All these findings suggest that miR-340 can be a potential prognostic marker or a therapeutic target.

## MATERIALS AND METHODS

### Cell culture and tissue specimens

Human breast cancer cell lines MDA-MB-231, MCF7, T47D, BT549, MDA-MB-468, normal breast cell line MCF-10A cells and HEK-293 cells were obtained from the Cell Bank of the Chinese Academy of Sciences (Shanghai, China) and cultured as previously described [40]. Specimens from 30 patients with primary breast cancer and the paired adjacent histologic normal tissues (> 2 cm distance from the margin of the resection) were immediately frozen in liquid nitrogen and stored at −80°C until use. This study was approved by the Institutional Review Board of TMUCIH and written consent was obtained from all patients. The tissues were snap frozen in liquid nitrogen within half an hour after the excision and stored at −80°C until use.

### Plasmids, miRNAs, and small interfering RNAs

miR-340 mimic, mimic-control, miR-340 inhibitor and inhibitor-control miRNA were chemically synthesized by RiBoBio (Guangzhou, China), as well as ZEB1 siRNA (5′-TGATCAGCCTCAATCTGCA-3′). The 3′-UTR of ZEB1 containing the putative miR-340 binding sites and the ORF of human ZEB1 was amplified by PCR. The PCR products were inserted into the pGL3-control luciferase reporter vector and pcDNA3.1 vector, respectively. The wild (P1) or Z-box-mutated (P2 to P8) miR-340 promoter were constructed to pGL3-promoter. All constructs were verified by DNA sequencing. Cells were planted in a 6-well plate before transfection. When the cells were 60% confluent, they were transfected with oligonucleotides (50 nM) by FuGENE6 transfection reagent (Promega, Madison, WI, USA) according to the manufacturer's protocol.

### Proliferation assays

MTT, plate colony formation and EdU assays were used to evaluate the ability of cell proliferation. For MTT assay, 24 h after transfection, 4 × 10^3^ cells were seeded in 96-wells plates per well. Cell viability was examined in the next 5 days. After incubation for indicated time, the cells were incubated with 20 μl MTT (5 mg/mL in PBS; Sigma-Aldrich, St Louis, MO, USA) at 37°C for 4 h. Then, the medium was removed and the formazan was dissolved in 150 μl of dimethyl sulfoxide (DMSO; Sigma-Aldrich). The absorbance was measured at 570 nm using a micro-plate auto-reader (Bio-Rad, Richmond, CA, USA).

For plate colony formation assay, 24 h after transfection, 500 cells were seeded in 6-well plate and cultured as normal. After about 15 days, the cells grew to visible colonies and were stained with crystal violet. The colonies were counted and compared with control cells.

The Edu assay were detected by EdU labeling/detection kit (Ribobio, Guangzhou, China) according to the manufacturer's protocol. Briefly, cells were incubated with 25 μM EdU for 12 h before fixation, permeabilization, and EdU staining. Cell nuclei were stained with Hoechst 33342 at a concentration of 5 μg/ml for 30 min. Then the cells were observed under a confocal laser scanning microscope.

### Transwell assays

The invasion ability of breast cancer cells *in vitro* was evaluated by Matrigel coated Transwell and Transwell inserts (BD Biosciences, San Diego, CA, USA). 1 × 10^5^ cells in 200 μl FBS-free medium were added in upper chamber of transwell and 10% FBS contained medium was added in lower chamber. After 16 hours, the cells were fixed by 4% paraformaldehyde and stained by Giemsa stain (Solarbio). Then the cells were observed under a microscope and the number of migrating cells was counted in five predetermined fields.

### Chromatin immunoprecipitation (ChIP) assay

ChIP assays were performed using a ChIP Assay kit (Upstate, Lake Placid, NY, USA) as previously described [40]. Briefly, cells were formaldehyde crosslinking and lysed. Then the lysate was sonicated and incubated with ZEB1 antibody or with control IgG overnight. A sample of “Input DNA” was collected before IP for normalization. After reversing the DNA–protein cross-links, chromatin DNA was purified and subjected to PCR analysis. ChIP DNA samples were analyzed with quantitative polymerase chain reaction (qPCR). Each ChIP DNA sample was compared to the appropriate Input DNA sample. PCR was carried out using primers specific for the ZEB1 binding region in the human mir-340 promoter (Z-box 1: forward 5′-CCTAGTCCAAAAGGTTCCC-3′ and reverse 5′-TCAGGCTCCTTTCACCTCT-3′; Z-box 2: forward 5′-GCCTGATCATAGTATGTGC-3′ and reverse 5′-GAAAGCTGAACAGGTAGCC-3′; Z-box 3: forward 5′-CCCTACTCCTTTTCCAGCT-3′ and reverse 5′-AGTAACTGAGACGGATCCC-3′).

### Luciferase assay

Cells were plated in 24-wells plate, cultured overnight, and cotransfected with pGL3-constructs with corresponding oligonucleotides. 48 hours later, luciferase activity was detected by using a dual luciferase assay kit (Promega) according to the manufacturer's recommendation.

### RNA extraction and quantitative real-time PCR

Total RNA was extracted from cells using RNAiso Plus (TakaRa, Dalian, China) following the manufacture's protocol. Reverse transcription was performed following protocol of PrimeScript RT reagent kit (TaKaRa). qRT-PCR was performed using SYBR Premix Ex Taq II (TaKaRa). β-actin was used as reference gene. Relative expression was quantified using the 2^−ΔCt^ method.

### Western blot and immunoflurescence

Total protein was extracted by lysing the cells with RIPA buffer and protease inhibitor. After denatured, proteins were run in the 10% SDS-PAGE gel and transferred to PVDF membranes. Membranes were blocked in 5% skim milk for 1 h at room temperature. Primary antibodies, vimentin (1:3000, abcam, Cambridge, MA, USA), E-cadherin (1:3000, abcam) and ZEB1 (1:1000, Santa Cruz Biotechnology, Santa Cruz, CA, USA) were incubated overnight at 4°C. After washed in TBST, membranes were incubated with anti-mouse or anti-rabbit antibodies (1:3000) at room temperature for 1 h. Protein bands were visualized by the ECL system (Millipore).

For immunofluorescence assay, cells were seeded in 24-wells plate. The next day, attached cells were fixed by 4% paraformaldehyde for 30 min, and penetrated by 0.5% Triton X-100 for 15 min, then blocked by 3% BSA for 1 h. Primary antibodies in 1% BSA, vimentin (1:300, abcam), ZEB1 (1:300, Santa cruz), were incubated overnight at 4°C. Then anti-mouse or anti-rabbit IgG FITC (1:500) were incubated in at room temperature for 1 h and then stained with DAPI. Finally, coverslips were observed under a fluorescence microscope.

### Statistical analysis

Each experiment was performed in triplicate. Data from experiments was expressed as mean ± SD. All statistical analyses were performed using SPSS18.0 software system for Windows (SPSS Inc., Chicago, IL, USA). Differences between groups were compared using student *t* test. *P* value less than 0.05 were considered significant.
